# Hemangioblastoma arising from duramater: Case report

**DOI:** 10.1097/MD.0000000000018076

**Published:** 2019-11-22

**Authors:** Bingyang Bian, Bei Zhang, Hongli Zhou, Junwei Tian, Zhuo Wang, Jiping Wang

**Affiliations:** a Department of Radiology, First Hospital of Jilin University; b Department of Nephrology, Second Hospital of Jilin University; c Department of Bone and Joint Surgery, First Hospital of Jilin University, China.

**Keywords:** dura mater, hemangioblastoma, meningioma, supratentorial neoplasms, diagnosis

## Abstract

**Rationale:**

Hemangioblastoma (HB) is a benign tumor that is typically located in the subtentorial region of the brain. HB that originates from dura mater is extremely rare.

**Patient concerns:**

Herein, we reported a single case of a patient who presented with dizziness and headache lasting for 1 year that progressively aggravated within 1 month.

**Diagnosis:**

After admission, the patient underwent head magnetic resonance (MR); a nodular long T1-T2 signal was found on the right side of parietal falx cerebri; the lesion appeared with high intensity on FLAIR and DWI, and with isointensity on ADC map. In addition, significant homogeneous enhancements were observed on the enhanced scan. According to clinical and imaging features, the lesion was diagnosed as meningioma. However, after performing tumor resection by craniotomy, the diagnosis of HB is clear. Additional pathological examination data were found: Ki-67(+1%), NSE(-), CD31(+), CD34(+), CD56(+), S-100(-), α-inhibin(+), Vimentin(-), EGFR-), GFAP(-), CK-pan(-), EMA(-), PR(-).

**Interventions:**

The mass with abundant blood supply was removed.

**Outcomes:**

Ten days after operation, the patient was discharged from hospital and no signs of recurrence were observed three months later.

**Lessons:**

To sum up, obvious high signal intensity in T2WI sequence and homogeneous enhancement are main characteristics that differentiate dural hemangioblastoma from meningioma lesion.

## 1 Introduction

Hemangioblastoma (HB) is a benign tumor of the central nervous system that commonly arises from the erythrocyte precursor. HB may occur sporadically or in association with von Hippel-Lindau disease (VHL) (25% of cases).^[1]^ It mainly arises in the cerebellar hemisphere and the cerebellar vermis, and the solid type accounts for about 20% of all HB. Tumor that originates from dura mater is extremely rare.^[2–4]^ Solid HBs can be easily misdiagnosed as intracranial tumors with abundant blood supply. In addition, the surgical removal of HB is extremely difficult. The safe resection of solid tumors involves preoperative embolization, circumferential dissection and wide exposure, which represent a challenge for the majority of neurosurgeons.^[5]^ Herein, we reported a single case of a patient with HB present in the cerebral falx with obvious uniform enhancement, which was misdiagnosed as meningioma.

## 2 Case presentation

A 70-year-old Chinese female who was experiencing headache for one month, was admitted to Our Hospital on April 5, 2018. The patient developed headache with no obvious inducement. No obvious relief from the headache was observed during the course of the disease. The pain was characterized by paroxysmal headache and was mainly located in the parietal with no pulsation and other accompanying symptoms. The headache occurred several times a day, lasting 3 to 5 minutes each time. In addition, the patient had no history of brain lesions.

**Figure 1:**
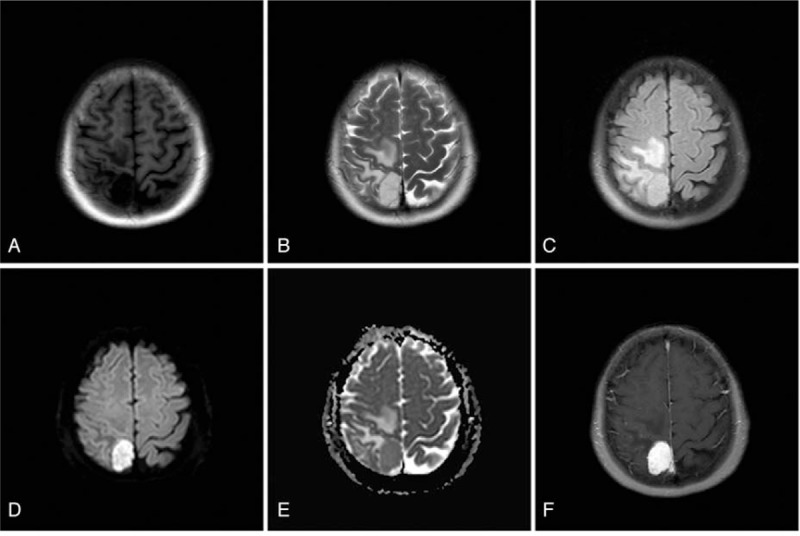
MR imaging features. (A and B) Nodular long T1 long T2 signals are located on the right parietal region. (C–E) The lesion showed high signal on FLAIR (C) and DWI (D) sequence, and low signal intensity on ADC map (E). Obviously homogeneous enhancement was also observed (F).

Consequently, the patient underwent neurological diagnostic examination; the size of both pupils were equal (3.0 mm in diameter); pupillary light reflex was sensitive; movement and sensation of limbs were normal; physiological reflex was present, and pathological reflex was not elicited. The remaining nerve system did not show abnormal activity. Next, the patient underwent head MR (on April 7, 2018). A nodular long T1 and T2 signal was found on the right side of parietal falx cerebri (Fig. 1A and B). FLAIR showed high signal intensity (Fig. 1C); DWI was slightly limited (Fig. 1D) and a low signal intensity was observed on diffusion coefficient (ADC) map (Fig. 1E). In addition, a significant homogeneous enhancement was also observed (Fig. 1F). The result of complete blood cell (CBC), routine urine test, renal function, liver function and blood biochemical parameters were all normal. The patient had no significant medical or family history. According to clinical and imaging features, the lesion was diagnosed as meningioma.

**Figure 2:**
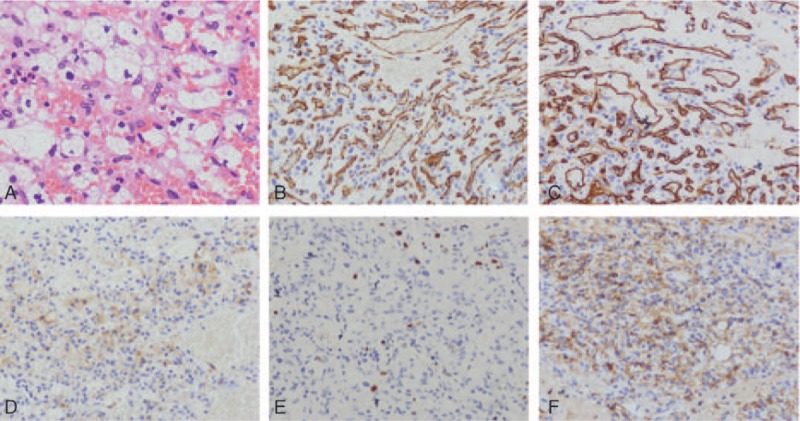
Immunohistochemistry. (A) Abundant capillaries and stromal cells can be seen under microscope. Tumor cells grow around the blood vessels and the cytoplasm is bright (HE × 400). (B–F) CD31 (+), CD34 (+), α-inhibin (+), Ki-67 (+1%), CD56 (+) (×200).

Since the patient had no contraindications for surgery, craniotomy was performed on April 12, 2018. During the operation, the tumor was observed within the dura surface. On the surface of dura, the parafalx tumor presented gray and hard with wide base, with abundant blood supply and complete capsule. There was no obvious adhesion between the lesion and the surrounding brain tissue. According to intraoperative condition, tumor was considered as dura mater tumor. Consequently, the resection of the tumor was performed. Postoperative pathological findings showed abundant capillaries and interstitial cells. The tumor cells were present around the blood vessels, and the cell cytoplasm was bright (Fig. 2A). Immunohistochemistry testing showed the following: Ki-67(+1%), NSE(-), CD31(+), CD34(+), CD56(+), S-100(-), α-inhibin(+), Vimentin(-), EGFR-), GFAP(-), CK-pan(-), EMA(-), PR(-). The outcome of the pathology showed the presence of hemangioblastoma (Fig. 2B–F).

The patient had a complete recovery of her preoperative symptomatology. Postoperative screening was performed using clinical, laboratory, ultrasound and body imaging scans and no association with VHL was observed. The patient was discharged on April 22, 2018 (ten days after the operation). The patient had a good compliance. Postoperative review was preformed three months after surgery. The patient's symptoms disappeared without complications and other adverse reactions. In addition, no residual tumor or recurrence was found on MR. Both the doctor and patient were very satisfied with the treatment effect. The patient was advised to make a MR review every three to six months.

## 3 Discussion

HB may occur sporadically in 75% of cases.^[1]^ The sporadic form is commonly solitary, and it frequently occurs in patients who are between 40 and 50 years old. Regarding the age presentation, it has been proposed that sporadic HB may arise from a two-stage mutation process.^[6]^ Herein, we reported a single case of a 70-year-old female, whose age characteristic was roughly consistent with the above inference.

Hemangioblastomas (HBs) are benign neoplasms (WHO grade I) that constitute roughly 2% of intracranial neoplasms.^[4]^ HBs often occur in the parenchyma of the brain, especially in the posterior fossa^[3]^, while tumors that originate from the dura mater are extremely rare. According to the WHO classification, the HBs derived from mesenchymal tissue are separately classified.^[2]^ HBs are highly vascular tumor types, most commonly detected in the posterior fossa and cervical spinal cord. HB rarely occur outside of the posterior fossa without VHL.^[7,8]^ Herein we reported a single case of HB connected to the falx with a broad base. Imaging characteristics included a solid, parietal parafalx mass that was homogeneously enhanced. During surgery, the lesion was identified as originating from dura mater.

Surgical removal of solid HB is a demanding and challenging task, with high morbidity and mortality.^[9]^ If HB is misdiagnosed as other intracranial tumors with abundant blood supply, such as meningiomas, this may increase the risk of surgery.^[9,10]^ Consequently, it is essential to differentiate HB from meningioma. In the present study we reported a case of supratentorial HB, which based on close association with the falx cerebrum and avid enhancement, strongly resembled a meningioma. HBs of the central nervous system (CNS) are best visualized on gadolinium-enhanced MRI. While MRI findings are not pathognomonic, HBs are typically seen on MRI as a cystic mass with an enhancing mural nodule, most commonly in the posterior fossa. The nodule is dark on T1-weighted images, bright on T2-weighted images, and homogeneously enhancing. For this case, the solid hemangioblastoma manifested low signal in T1WI and high signal in T2WI. Meningioma is a common intracranial primary tumor. MRI is an effective means of diagnosing meningioma. Typical meningioma has equal or slightly longer T1 and equal or slightly longer T2 signal with homogeneous enhancement. In fact, the differences in T1 and T2 signals can be one of the main identifications between them. Flow void of the vessels can be more commonly found in HB. Although the lesion is similar to those observed in meningiomas, there are clues that can be identified in the imaging findings.

HB does not usually recur after definitive treatment.^[8]^ After surgical excision of the tumor was performed, there were no other follow-up treatments. Furthermore, no signs of recurring were found in the first review.

## 4 Conclusions

Although uncommon, HB is a tumor that should be considered when dealing with solid vascularized tumors, even in relation to the parietal falx cerebri mimicking meningioma. This case can be used as a useful supplement to the imaging findings of solid HB.

## Author contributions

**Resources:** Bingyang Bian, Hongli Zhou.

**Writing - Original Draft:** Bingyang Bian, Bei Zhang, Junwei Tian.

**Methodology:** Zhuo Wang.

**Software:** Zhuo Wang.

**Writing - Review & Editing:** Jiping Wang.
